# Development of Individualized Cognitive Augmentation Regimen for Elderly (iCARE): Preliminary findings of a caregiver‐driven cognitive training for dementia in India

**DOI:** 10.1002/alz.090610

**Published:** 2025-01-09

**Authors:** Aparna S Kanmani, Keshav K Janakiprasad, Sivakumar PT, PS Mathuranath

**Affiliations:** ^1^ National Institute of Mental health and Neuroscience ‐ HOSPITAL, BENGALURU, Karnataka India; ^2^ National Institute of Mental Health and Neurosciences (NIMHANS), Bengaluru, Karnataka India; ^3^ Departmet of Psychiatry, National Institute of Mental Health and Neurosciences (NIMHANS), Bengaluru, Karnataka India; ^4^ National Institute of Mental Health and Neurosciences, Bengaluru, Karnataka India

## Abstract

**Background:**

With increased for promoting neuroplasticity in older adults through Cognitive training (CT), the study aimed to develop culturally relevant caregiver‐driven model of CT for dementia called the Individualized Cognitive Augmentation Regimen for Elderly (iCARE).

**Method:**

The study has three phases‐

1. Development Phase‐ Included a) literature review, b) item generation, c) expert rating, d) field trial (n = 3), and e) feedback and modification. The module is designed as a flip book primarily targeting eight cognitive domains. A unique method of empowering caregivers (family or professional) as co‐therapists was used to deliver the intervention on adequate receiving training.

2. Pilot Phase ‐ A quasi‐experimental research design was used to recruit patients (n = 8) diagnosed with mild‐moderate dementia (DSM V criteria) from a clinical setting. The eligible participants were engaged as dyads (1 patient + 1 caregiver) and received either Treatment as usual (TAU) or iCARE program (1:1 ratio) for a duration of 5 weeks. They were assessed at three time points (baseline, post‐intervention, and 1‐month follow‐up).

3. Main Phase – The modified program was administered on 32 dyads (16 Intervention, 16 TAU) meeting the inclusion and exclusion criteria using the same protocol. The NIMHANS neuropsychological battery for elderly (NNB‐E) was used to determine the neuropsychological profile of the patient along with rating scales to track cognitive symptoms, quality of life, activities of daily living, behavioural/psychological symptoms, caregiver health and burden.

**Result:**

The study revealed positive results in task‐related performance, outcome variables, and functional changes. The neuropsychological profiles post‐iCARE demonstrated improvement in attention, working memory, fluency, verbal recognition and logical and visual memory. Compared to TAU, enhancement was noted in quality of life, independence in daily activities, and symptom severity. Caregivers also reported advancement in confidence, error detection, self‐correction, communication and insight. The caregivers' high rating for feasibility of iCARE make it suitable for Indian population. Qualitative evidences of the same shall be presented.

**Conclusion:**

The iCARE program intends to support families learn a better‐quality home‐based management of dementia. The method of using interactive materials and caregivers as active co‐therapists is recommended to address cognitive decline in dementia.

